# Retraction: Neurotoxicity Induced by Bupivacaine via T-Type Calcium Channels in SH-SY5Y Cells

**DOI:** 10.1371/journal.pone.0238400

**Published:** 2020-08-25

**Authors:** The PLOS ONE Editors

Following publication of this article [1] concerns were raised regarding images in Figs 5 and 7, as detailed in an Expression of Concern published 12 September, 2019 [2]. The authors have carried out further repeat experiments. However, in view of the unavailability of the original underlying data, the *PLOS ONE* Editors consider that the issues raised cannot be fully resolved by the provision of repeat experimental data. The corresponding authors requested the article's retraction.

In light of the unresolved issues with Figs 5 and 7, and in line with the corresponding authors’ request, the *PLOS ONE* Editors retract this article [1].

XW, SX, QZ, and HLiang agreed with the retraction. XW, SX, and QZ, and HLiang stand by the article's findings. HLiu, CY, and HW either could not be reached or did not reply directly.
